# Synthesis, Characterization and Histopathological Study of a Lead-Based Indian Traditional Drug: *Naga Bhasma*

**DOI:** 10.4103/0250-474X.62232

**Published:** 2010

**Authors:** S. K. Singh, D. N. S. Gautam, M. Kumar, S. B. Rai

**Affiliations:** Laser and Spectroscopy Laboratory, Department of Physics, Banaras Hindu University, Varanasi-221 005, India; 1 Department of Rasa-Shastra (RGSC), Institute of Medical Sciences, Banaras Hindu University, Varanasi-221 005, India; 2 Department of Pathology, Institute of Medical Sciences, Banaras Hindu University, Varanasi-221 005, India

**Keywords:** Ayurvedic drug, *Naga bhasma*, nano particles, traditional medicines

## Abstract

The aim of the present study is to prepare and characterize *Naga bhasma* on structural and elemental basis to address the role of the raw materials used during the preparation, compound form of the lead *bhasma*, nature (crystalline/amorphous) and crystallite/particle size of the drug. The study also covers the toxicological effect of the drug on albino rats. It was found that drug contains lead in nano-crystalline (~60 nm) lead sulfide form (Pb^2+^) associated with the organic contents and different nutrient elements coming from the herbs used during the preparation. *Naga bhasma* prepared was found to be totally safe in histopathology study on rats at a dose of 6 mg/100 g/day. The different characterization techniques used present a role model for the quality control and standardization of such kinds of herbo-metallic medicines.

Herbal medicines have been important ingredients in human life since earliest times and it has recently acquired increasing importance due to its supposedly harmless nature and effectiveness[1]. Different alternative systems of medicines, including Ayurveda make use of herbal preparations for their curative effects. Use of metallic herbal preparations (also called *Bhasma*), in which a process termed *bhasmikarana* is used to prepare the drug, is unique to the Ayurveda. It is believed that *bhasmikarana* process converts the metal into its specially desired chemical compound which eliminates the toxicity of the metal and has the necessary medicinal benefits[2,3]. Ayurvedic texts provide a list of tests for the efficacy of the *bhasmikarana* process. The tests which are essentially qualitative ensure that the resulting drug is very fine (small grains), has no metallic shine and does not alloy with silver even at higher temperature to which it was subjected[4–7]. However, these qualitative tests do not provide any quantitative information about the composition and the structure of the final drug. For any drug containing heavy metals (for example lead, mercury), such structural information is an absolute necessity[8,9].

*Naga bhasma* (which includes lead and different herbs) is one of such metallic preparation used in various diseases such as diarrhea, spleen enlargement and diabetes[4]. Though some research work[10,11] has been carried out on the different curative applications of *Naga bhasma* but none of them give the detail on the elemental and structural composition of the drug *Naga bhasma* which is an essential requirement to discuss its non-toxicity and therapeutic value.

The aim of the present study was to characterize the *Naga bhasma* on elemental and structural point of view. The compositions of medicinal herbs (used during the processing of the *bhasma*) have also been investigated. X-ray diffraction (XRD) and transmission electron microscopy (TEM) were used for the detection of compound type, crystalline/amorphous nature and the crystallite size present in *Naga bhasma*. It is believed that at high temperature during the processing of *bhasma* possibility of organic compounds are rare. However it is found that organic molecules derived from the herb remain present in *bhasma* and probably act as the coating material on the metal compound used in the *bhasma*[2]. Infrared (IR) spectra of the raw materials used in processing of the drug and the *bhasma* were studied to ascertain the presence of organic matter in the finally prepared drug. Concentrations of the different elements present in the raw materials and *Naga bhasma* were measured using energy dispersive X-ray analysis (EDAX) and atomic absorption spectroscopy (AAS). The histopathological studies have also been carried out on Charles Foster (CF) rats.

## MATERIALS AND METHODS

### Preparation of *Naga bhasma*:

Processing of the *Naga bhasma* was done according to the “*Shastiputa Naga bhasma*” process listed in the “*grantha Ananda Kanda* 2/6/25-28”. Raw materials were obtained from the pharmacy of the Institute of Medical Sciences, Banaras Hindu University, India. Lead so obtained was purified through sublimation. Lead metal was melted in iron ladle and poured into a vessel containing lime water (called *Curnodaka,* decoction strength 4.31 g/l) and filtered. The process was repeated seven times with fresh lime water each time. The same process was done with decoction (decoction strength 250 g/l) of *nirgundi* (*Vitex negundo* Linn.) leaf and turmeric (*Curcuma longa* Linn.) powder again for seven times.

In the first *puta* (step), the purified lead thus obtained was melted with equal amount of *manahsila* (AS_2_ S_2_) and a small amount of *Chichiri* (*Plectranthus coesta* L Her.) herb (root, stem, leaves, flower and fruit all parts were used). Melted lead was stirred constantly with *Neem* (*Azadirachta indica* A Juss.) stick till it becomes dried powder. After cooling, powder is triturated with the juice of *Vaasa* (*Adhatoda vasica* Nees.) leaf. Small pellets were made and dried in shade. Dried pellets were packed airtight in two earthen pots one above the other (called *Sharav samput*). Finally the pots were subjected to heat in the electric furnace at 600° in aerobic condition. This was the first *puta* (step) *Naga bhasma* sample.

Sample thus obtained was used in the next step. In rest of the each steps (remaining 59 step), *manahsila* was added in 1/20^th^ proportion to the prepared *bhasma* with juice of *Vaasa* and subjected to heat treatment. The process was repeated sixty times to get the finally prepared *Naga bhasma.* The final product in the form of the pellets were taken out of the earthen pot and powdered. The powdered material was packed in airtight containers. Total of three batches were prepared in a six month time.

### Physico-chemical characterization:

Powdered *bhasma* was characterized by powder X-ray diffraction (XRD) using a Philips 1710 X-ray diffractometer with CuKα radiation (λ=1.5405 A°). We have also used transmission electron microscope (TEM) Philips, CEM, CM-12 for characterization of nano structure and the phase of the sample. X-ray photoelectron spectrum (XPS) measurement was performed on ESCLAB MKII instrument, using none monochromatized MgKα X-ray as the excitation source. For IR spectra, powdered samples were mixed in KBr to make translucent pellet. The spectrum was recorded from 400-4000 cm^−1^ region using FTIR spectrophotometer, Spectrum RXI (Perkin Elmer). EDAX attached to TEM was used for the detection of various elements in the sample. For quantitative detection of trace metals in parts per million (ppm) an atomic absorption spectrophotometer (AAS. model no. 2380, Perkin Elmer) was utilized. The detail of the sample preparation and the method followed in AAS measurement is given in references 1 and 12.

### Histopathological studies:

Histopathology study of skin, small intestine, pancreas, testis, brain, lung, kidney, and liver was performed on CF rats. For this study, fifteen CF rats (both male and female of nearly same weight) were distributed in three groups each group contained 5 animals of both sexes of weight varying from 100-175 g. The first group of animals were kept as control (untreated animals). The second group of animals were treated by crude lead (6 mg/100 g of body weight per day) and the third group of animals were treated by *Naga bhasma* (6 mg/100 g per day). The dose is based on the human dose. According to the human dose (i.e. 125 mg/50 Kg for a healthy human) the dose for animal is 0.25 mg/100 g per day. A higher dose (adopted in the animal experimentation) of 6 mg/100 g of body weight per day has been tried for the toxicity study. These experiments were carried out continuously for 40 days. For histopathological study, samples of the organs of all the three groups were collected, fixed with 4% formaldehyde, dehydrated with ascending grades of alcohol and then embedded in paraffin (melting point 58-60°). From these blocks, 0.5 μm transverse sections were cut on rotary microtome and the sections were stained by haematoxylin and observed under microscope on different magnifications. The histopathological studies were carried out with due permission of the ethical committee for research animal experimentation of the University under the reference RAC/RES-MED 34, dated 21.11.96. The permission was taken much before but the final experiments were done in 2001. Histopathological study was carried out at the Department of Pathology, IMS, BHU.

## RESULTS

### XRD, TEM and XPS analysis:

**Fig. 1:**
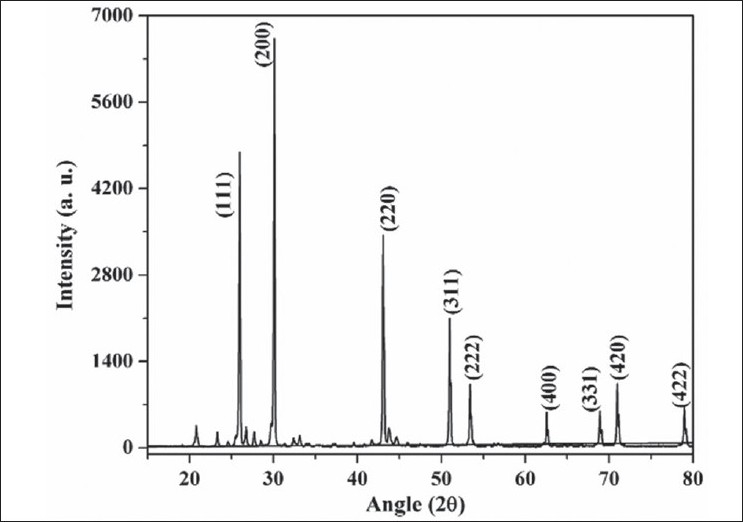
XRD pattern of *Naga bhasma*. The sharp diffraction peak in the XRD pattern shows the nanocrystalline nature of the PbS phase present in the *Naga bhasma*.

X-ray diffraction pattern of the *Naga bhasma* is shown in fig. 1. Presence of sharp diffraction peaks shows the highly crystalline nature of the drug. The diffraction peaks at angle 2θ = 25.96, 30.07, 43.05, 50.97, 53.40, 62.53, 68.87, 70.96 and 78.91° corresponding to the (111), (200), (220), (311), (222), (400), (331), (420) and (422) planes, respectively (JCPS File No.: 05-592) confirms the presence of the single lead sulfide phase corresponding to the metal lead. No diffraction peak due to the oxide phase of the lead was detected. The crystallite size was calculated from the XRD pattern following the Scherer equation t = (λ × 0.9)/(β × Cosθ), where, t is the crystallite size for (h k l) plane, λ is the wavelength of the incident X-ray radiation (CuK_α_ (0.154056 nm)), β is full width at half maximum (FWHM) in radians and θ is the diffraction angle for (h k l) plane. The crystallite size calculation was done corresponding to the diffraction peaks (111), (200) and (220) planes. The above equation yields t = 60±4 nm. Thus the XRD study concludes the presence of nano-crystalline structure of the drug.

**Fig. 2:**
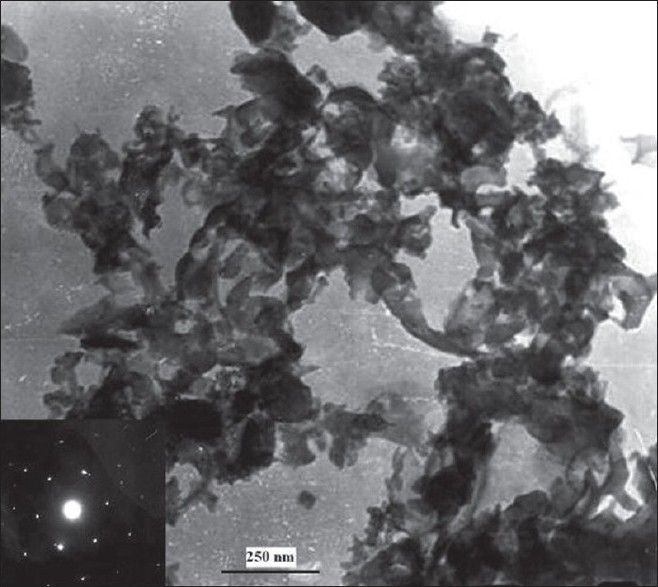
TEM image of *Naga bhasma*. Transmission electron microscopic (TEM) image with selected area electron diffraction (SAED) pattern of *Naga bhasma* showing the presence of micro-sized particle

TEM image along with selected area electron diffraction (SAED) pattern of the sample is shown in fig. 2. Fairly bright spots in the SAED pattern again support the highly crystalline nature of the sample. The TEM image of the drug sample shows spongy like structure with the irregular particle size in the submicron range. The reason is the use of the organic materials. Due to the organic materials from the herbal source in the preparation of the *bhasma* and heat treatments, the nano size crystallite get agglomerated and give rise to the micro sized particles. These studies confirm that the *bhasma* are nano-crystallte with submicron size particle.

**Fig. 3:**
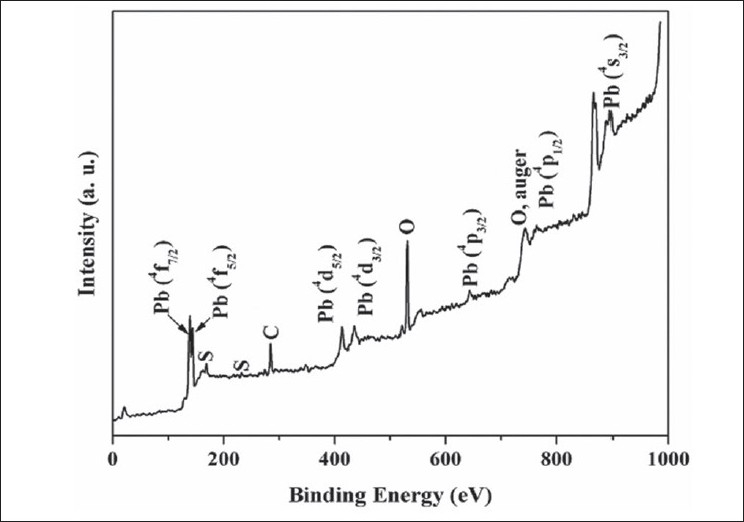
XPS spectrum of *Naga bhasma*. Typical X-ray photoelectron spectroscopy (XPS) survey spectrum of the drug *Naga bhasma* showing the presence of different elements on the surface of the drug

The XPS analysis provides valuable information for the surface state of the drug sample. Fig. 3 shows a typical survey spectrum of the drug *Naga bhasma*, confirming the presence of lead and sulfur. In addition to these, it also shows the presence of carbon and oxygen peaks. Although our EDAX study (described later) shows the presence of the Mg, Ca, and Fe, yet, these ions were not observed in XPS analysis, indicating their absence on the surface. Presence of C and O, which are the building blocks of the organic materials, on the surface of the drug by XPS supports the idea of coating of organic molecules on the surface of the metallic compounds. High resolution spectra at Pb core level showed the presence of the peaks at 139.28 eV and 145.32 eV corresponding to Pb(^4^f_7/2_) and Pb(^4^f_5/2_) while S core level appeared at 169.08 eV corresponding to S (^2^P_3/2_), respectively for PbS phase. Thus the XPS analysis also confirms the presence of PbS phase on the surface of the drug *Naga bhasma*.

### Infrared analysis:

**Fig. 4:**
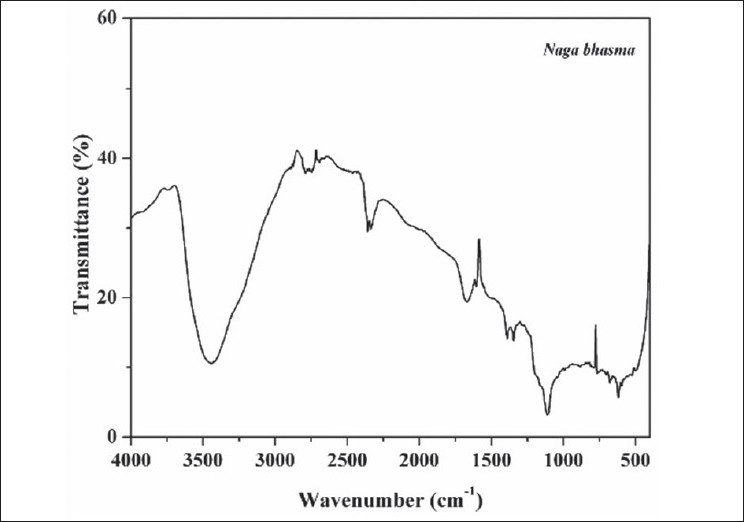
FTIR spectrum of *Naga bhasma*. Fourier transform infrared spectrum of the drug *Naga bhasma* showing the presence of organic molecule in the drug

FTIR spectrum of the sample is shown in fig. 4. Spectrum of the sample shows large no of well defined peaks in 400-2000 cm^−1^ region along with peaks in higher frequency region. Broad peak at 3440 cm^−1^ is due to the OH^−^ (due to moisture). Plant materials used in the preparation contains several organic molecules such as turmeric powder which contains curcumin and curcumino. *vaasa* contains alkaloids vasicine, adhatodinine and several other types of molecules in traces. *Neem* contains azadirachtin and derivatives basically terpins and limonoids and *nirgundi* leaf contain flavonoids, alkaloids and terpinoids[13–15]. All of these molecules give well defined IR peaks due to them or their transform (due to heat treatment) in the fingerprint region (400-2000 cm^−1^). These peaks are also present in the *bhasma* (fig. 4). Thus from the FTIR spectra it is concluded that the finally prepared *bhasma* is associated with the organic macromolecules from herbs used in the preparation. These organic molecules certainly play an important role to increase the efficiency of *bhasma*. Attempt to find out their activity will certainly improve the understanding of *bhasma*.

### EDAX and AAS analysis:

**Fig. 5:**
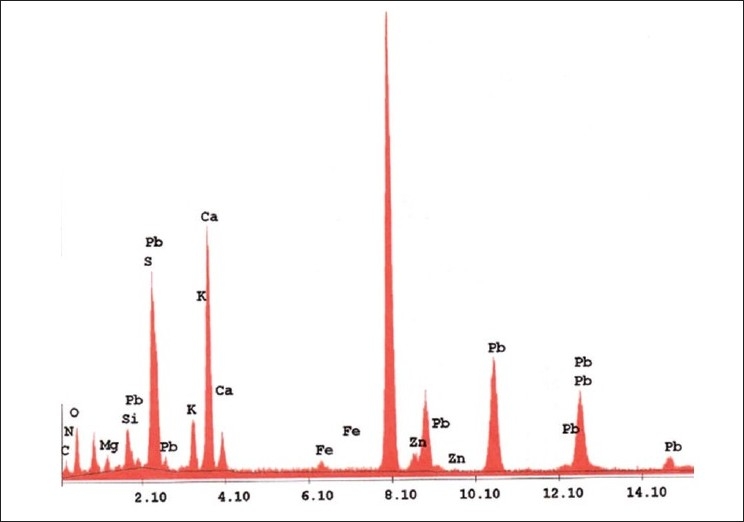
EDAX of *Naga bhasma*. Energy dispersive X-ray analysis (EDAX) of the *Naga bhasma* showing the presence of different element in drug.

In addition to the metal Pb used in the drug, other metals are also expected in the drug which enters in it during its pharmaceutical processing. EDAX is used to detect the elements present in considerable amount (quantitative determination of bulk elemental composition) where as the AAS method is used to detect the elements present in traces. Fig. 5 represents the different elements present in the *bhasma* sample observed in EDAX analysis. Table 1 lists the chemical compositions of *Naga bhasma* using EDAX and Table 2 lists the trace metal composition of *Naga bhasma* along with the other herbs (used as raw materials in the preparation of the *Naga bhasma*) using atomic absorption spectroscopy. *Bhasma* is found to be rich in Mg, Ca and Fe which are most essential for a healthy metabolism and responsible for the absence of side effects as regards stomach lesions. The electrolytic elements Na and K responsible for maintaining normal fluid balance inside and outside cells are also found to exist in large concentration. Zn which is useful for proper growth and to increase the immunity of the body is also found in sufficient concentration. All these elements taken from herbs act as the essential elements to increase the efficiency of the drug and seem to be additional supplement in cure of the disease. Other heavy metals (Cd, Cr, Cu and Ni) found are in small amount and well within the limit of safety recommended by world health organization (WHO) for daily intake. Lead was found to be high enough but as we have already found via structural analysis that lead is present in the lead sulfide form (least toxic). From the Table 2 it is clear that medicinal herbs used in preparation of *bhasma* are very rich in essential nutrients such as Ca, Fe, Mg, and Zn. Thus elemental analysis shows that the nutrient element present in *bhasma* sample are due to herbal sources and may be called as bio-available.

**TABLE 1:** MACRO ELEMENT COMPOSITION OF *NAGA BHASMA* BY EDAX ANALYSIS

Elements	Weight %^a^	Atomic %^a^
C	21.81	30.37
N	11.60	13.85
O	44.70	46.73
Mg	2.76	1.90
Si	3.88	2.31
S	0.98	0.51
K	1.83	0.78
Ca	7.28	3.04
Fe	0.18	0.05
Zn	0.26	0.07
Pb	4.70	0.38

a Based on ZAF (atomic number, absorption, fluorescence) quantification (standardless).

**TABLE 2:** TRACE ELEMENT COMPOSITION (IN PPM) OF *NAGA BHASMA* AND HERBS BY AAS

Element	*Naga bhasma*	*Vaasa* leaf	*Neem* stem	*Nirgundi* leaf
Ca	283±23.96	128±11.48	98±08.68	642±57.78
Mg	62±08.06	56±07.78	24±02.88	176±08.80
Fe	19±01.04	---	01±0.16	234±28.08
Cu	02±0.32	0.42±0.089	0.35±0.073	03±0.54
Na	08± 1.04	---	---	35±04.55
K	42±3.72	---	---	13±01.04
Zn	10±1.40	1.72±0.27	0.95±0.218	14±02.24
Mn	05±0.25	2.86±0.37	51±9.69	42±08.82
Ni	0.47±0.09	---	0.32±0.083	---
Cr	0.35±0.06	0.15±0.03	0.07±0.012	1.02±0.29
Cd	0.04±0.01	0.02±0.006	0.02±0.003	0.07±0.02
Pb	740±35.38	0.11±0.026	0.39±0.62	0.21±0.03

Values are arithmetic mean ± standard deviation of three determinations in each case. ppm- parts per million, AAS- atomic absorption spectroscopy

### Histopathological study of vital organs of Charles Foster rats:

**Fig. 6:**
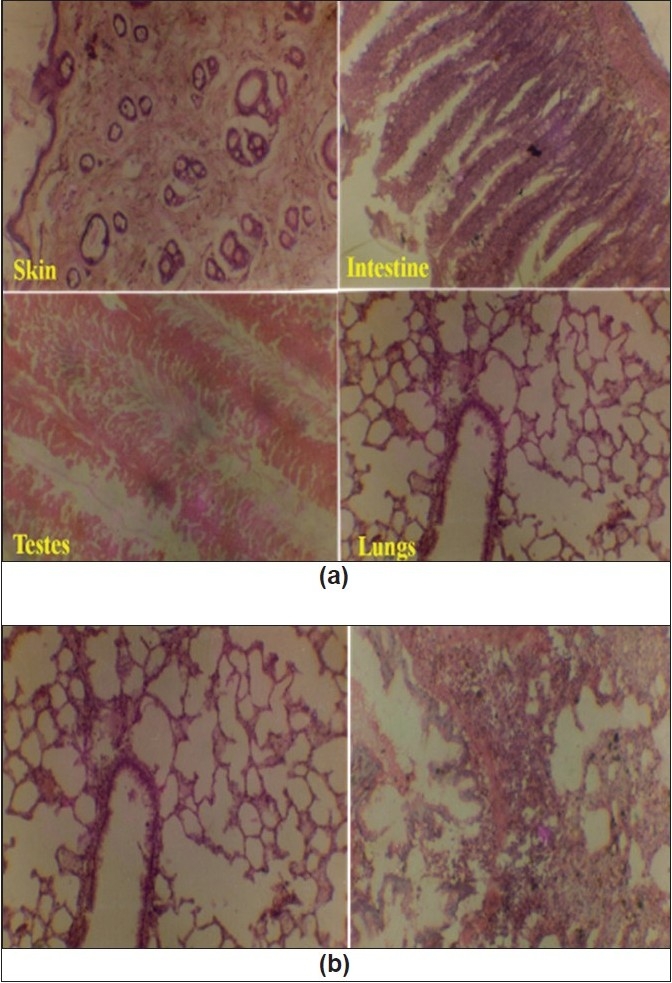
Images of the different organs of the albino rats. (a) Images of the different organs of the Charles Foster rats treated with *Naga bhasma* showing no effect (b) Images of the lungs of the *Naga bhasma* treated (left) and crude lead treated (right) albino rat. Pneumonic extensive changes in lung of crude lead treated rats are clearly visible

Histopathological studies of the skin, small intestine, pancreas, testis, brain, lung, kidney and liver were performed after 40 days of continuous crude lead treatment (6 mg/100 g/day), *Naga bhasma* treatment (6 mg/100 g/day) and in untreated animals. Fig. 6 (a) shows the images of the different organs of the albino rats treated with *Naga bhasma*. No significant changes were observed in histology as well as normal anatomy of the skin, small intestine, pancreas, testis, brain, lung, kidney and liver in *bhasma*-treated and untreated groups of animals, while in case of crude lead treated group of animals significant changes in certain organs were observed. For example, Fig. 6(b) shows the images of the lungs of the *Naga bhasma*-treated (left) and crude lead-treated (right) rats. Pneumonic extensive changes in lung of crude lead treated rats are clearly visible. Similarly cloudy swelling and congestions in kidney, congestions in spleen and liver and astrocytic proliferation in cerebrum (brain) were also seen in lead treated animals.

Preliminary studies in our lab show no significant changes in super oxide dismutase activity (SOD), protein, alkaline phosphatase, SGOT (serum glutamate oxalo transaminase), SGPT (serum glutamate pyruvate transaminase), creatinine, lipid peroxidase, serum urea in *Naga bhasma-*treated and untreated rat groups. Thus, histopathological studies show that *Naga bhasma* is non-toxic (6 mg/100 g/day), while crude lead (6 mg/100 g/day) is highly toxic. Thus it appears that in *bhasmikarana* process the crude lead is converted into *Naga bhasma,* which is found to be non-toxic at lower dosages.

## DISCUSSION

Preparation procedure of the *bhasma* is very elaborate. The starting steps are basically to quench the melted lead into different environment (due to different herbal juice) to interact the metal to the organic parts of the herbs. Further grinding and heating of the material several times in presence of some herbal juices leads to the generation of specific compound form of the elemental lead of highly crystalline nature. Submicron size particle of the sample may be attributed to the grinding of raw materials for a long duration (similar to the Top-Down approach of the formation of nanostructure materials in modern nanotechnology) as well as the heat treatment which causes the change in the chemical nature of the raw materials.

Further, it is a general belief that organic molecules get burned out at the processing temperature of the *bhasma* (i.e. above 400° in most of these kinds of preparations). However the infrared analysis shows the presence of organic matter in the sample because the inorganic phases present (such as PbS) in the drug do not give vibrationl peaks in 400-4000 cm^−1^ in the IR spectrum. Thus, this could be attributed due to the formation of organometallic complexes in the drug sample that can sustain even at high processing temperature of the *bhasma*. Purification (with lime water, decoction of *nirgundi* leaf, turmeric powder and *chichiri*) and stirring with *neem* stick associates the organic macromolecules with the lead sample and give rise to the formation of different organometallic compounds along with lead sulfide. *Neem* (stick) possesses bitter juice of astringent quality which is beneficial in diabetes. The process is further facilitated by the juice of *vaasa* leaf used during trituration for several hours. Thus it may be hypothesized that the *Naga bhasma* acts as the carrier of the medicinal property of *nirgundi*, turmeric, *vaasa* and *neem*. *Nirgundi* leaf which is used as analgesic, antiinflammatory, carminative, diuretic, anthelmintic. Turmeric powder which exhibit a wide range of biological activity e.g. antibacterial, antiinflammatory, hypolipidemic, hepatoprotective and *vaasa* leaf which checks *pitta*, *sleshma*, blood diseases. *Neem* stick is beneficial in diabetes[16–17]. Presence of carbon and oxygen on the surface of the drug by XPS analysis also supports the idea of the association (coating) of organic molecules on the surface of the metallic compounds. These macro molecules associated with the *bhasma* certainly play an important role in increasing the efficacy and efficiency and in making these drugs biologically assimilable. Effort in the detection and role of these organic molecules is highly needed.

Elemental analysis shows the presence of different nutrient elements in considerable trace amount. The specific role of these elements in the *bhasma* is not yet very clear. It will also be important to study which step(s) of the complex preparation process, imparts the unique property in the *bhasma*. Histopathological study in the article is preliminary and shows the non-toxic nature of the drug at low dosages. An extensive study is needed for the complete pharmacokinetic study on the animal system.

Although, these experimental observations will help in the quality assurance and standardization of *bhasma* yet some more work is needed in this direction to find out more simplified methods which can be used for routine testing in the Ayurvedic industry.
